# Natural History of Visual Acuity and Microperimetry-Based Functional Outcome Measures of the Macula in Patients with Geographic Atrophy: A Retrospective Chart Review Study in Germany

**DOI:** 10.3390/jcm13133959

**Published:** 2024-07-06

**Authors:** Paul Kohlhas, Alaa Din Abdin, Wissam Aljundi, Ann-Isabel Mattern, Machteld Devenijn, Kathrin Borchert, Andreas Fricke, Tammo Viering, Jürgen Wasem, Berthold Seitz, Hakan Kaymak

**Affiliations:** 1 Department of Ophthalmology, Saarland University Medical Center, Kirrberger Straße 100, 66424 Homburg, Germany; alaadin.abdin@uks.eu (A.D.A.);; 2 Internationale Innovative Ophthalmochirurgie GbR (I.I.O.), Theo-Champion-Str. 1, 40549 Düsseldorf, Germanydr.h.kaymak@gmail.com (H.K.); 3 Xcenda GmbH, Part of Cencora, Lange Laube 31, 30159 Hannover, Germany; 4 Department for Health Care Management, University Duisburg-Essen, Thea-Leymann-Str. 9, 45127 Essen, Germany; 5 Gottfried O.H. Naumann-Institute of Epidemiology and Prevention of Myopia, Saarland University, 66424 Homburg, Germany

**Keywords:** age-related macular degeneration, geographic atrophy, natural disease progression, visual impairment, microperimetry, scotoma

## Abstract

**Background/Objectives:** Geographic atrophy (GA) is an advanced form of age-related macular degeneration (AMD) leading to the progressive and irreversible loss of visual function. Characteristics of GA include atrophic lesions resulting from the loss of photoreceptors, retinal pigment epithelium, and choriocapillaris. During GA progression, atrophic lesions typically advance from the macular periphery to the center, affecting foveal light sensitivity and visual acuity. This study analyzed changes in light sensitivity and visual acuity during the natural course of GA progression using the topographic analysis of structural and functional changes based on Early Treatment Diabetic Retinopathy Study (ETDRS) charts, multimodal imaging, and microperimetry assessment. **Methods:** Medical chart data of GA patients between 2014 and 2022 from the Internationale Innovative Ophthalmochirurgie GbR (I.I.O.) research center (Düsseldorf, Germany) were retrospectively analyzed. All patient eyes fulfilling the phase 3 OAKS study inclusion criteria were included and followed up for 60 months. The imputation of missing measurements and dropouts was performed by linear mixed models. **Results:** A total of 20 GA eyes from 13 GA patients were included in the study. At the index, 53.8% of patients had bilateral GA, with 70.0% of the eyes showing multifocal GA and 30.0% subfoveal encroachment (SFE). A total of 35.0% of the eyes had 2–5, and 15.0% over 20, areas of atrophy. Over time, the GA lesion size increased from 6.4 mm^2^ to 11.8 mm^2^ (1.08 mm^2^/year). After an average observation time of 2.9 years, 78.6% of the initially unaffected study eyes developed SFE. The percentage of study eyes without visual impairment decreased from 55.0% to 30.0%, with mean normal-luminance best-corrected visual acuity (NL-BCVA) reducing from 63.7 to 55.7 ETDRS letters. The share of absolute scotoma points in microperimetry assessment increased from 15.7% to 43.5% while overall average macular sensitivity declined from 15.7 dB to 7.4 dB. **Conclusions:** The substantial deterioration of macular outcomes and visual function was comprehensively detected. The results were a documentation of structural and functional aspects of the natural progression of GA for a 60-month follow-up, providing a typical outline for AMD patients with GA.

## 1. Background

Age-related macular degeneration (AMD) is an acquired degeneration of the central retina, caused by pathological subretinal deposits, leading to progressive and irreversible visual impairment [1]. While being the most common cause of blindness in developed countries, AMD accounts for about 9% of global blindness. In comparison to Asians and Africans, with around 7.5%, the share of AMD patients within populations of European ancestry is significantly higher at around 12% [2,3]. Up to 50 million people are affected by AMD worldwide, and in Germany, approximately 0.37 million were diagnosed with exudative and 0.64 million with non-exudative AMD (including early, intermediate, and atrophic late AMD) in 2021 [2,4,5].

AMD can be classified by clinical manifestations into early (small drusen, without retinal pigment epithelium (RPE) abnormalities), intermediate (drusen > 125 µm and RPE abnormalities), and advanced stages, characterized by either the development of macular neovascularization (MNV) or geographic atrophy (GA) or both [6,7]. The advanced stages occur at similar rates [8]. 

A sign of GA is the occurrence of demarcated and depigmented lesions as a result of the degeneration of photoreceptors, the RPE, and underlying choriocapillaris [1,9]. Typically, the first GA lesions occur in perifoveal regions, subsequently coalescing and expanding towards the fovea [10]. The fovea as the most important macular region for central vision may remain unaffected until late stages (‘foveal sparing’); thus, central visual acuity might be relatively preserved in early stages of the disease [11]. The eventual foveal encroachment is associated with pronounced vision loss [12].

While effective therapies have been established for neovascular AMD, all previous attempts to prevent or slow down the progression of GA were not effective [13,14]. Recently, the pathogenetic role of a dysregulated complement system has been recognized and the inhibition of the complement system has emerged as a new therapeutic approach [15,16]. Indeed, recent studies have demonstrated the efficacy of intravitreal complement inhibitors, especially the complement C3 inhibitor peptide Pegcetacoplan, in reducing the growth rates of GA lesions [17]. Pegcetacoplan efficacy was recently confirmed by results of two phase 3 clinical trials, OAKS (NCT03525613) and DERBY (NCT03525600) [18,19,20,21,22]. 

The present study was based on data from medical charts and was assigned to investigate the natural development of functional endpoints with a specific focus on visual acuity and microperimetry assessment. Currently, the only phase 3 trial with available microperimetry data in which GA patients were treated with complement inhibitors is the OAKS study. The selection of the patients and variables of the present study was thus based on the protocols of the OAKS study in order to achieve comparability and to address potential patients for possible future treatment. Further objectives of the present study were to assess the longitudinal development of functional patient-relevant endpoints of the macula and visual impairment and to evaluate their clinical relationship with anatomical aspects such as subfoveal encroachment.

## 2. Methods

### 2.1. Study Design and Setting

Medical chart data were retrospectively analyzed from AMD patients with GA who presented to the I.I.O. research center between 2014 and 2022 and met the inclusion criteria with at least one eye. All patient eyes were included that met the inclusion criteria of the OAKS study. The inclusion and exclusion criteria were adopted to align with the requirements of the present study [18,21]. The first on-site microperimetry assessment was defined as index event with a required follow-up period of at least 2 years. The available follow-up time period for study eyes was of up to 60 months. The relatively low number of study eyes was due to the strict orientation to OAKS study criteria, leading to less statistical power but better comparison to OAKS and thus better transferability of results.

Retinal light sensitivity including detection of scotoma and fixation stability was measured by microperimetry (macular integrity assessment (MAIA), CenterVue^®^, Padua, Italy) and macular topography via spectral-domain optical coherence tomography (SD-OCT, Cirrus, Carl Zeiss^®^, Jena, Germany). 

Assessments of MAIA, OCT, and ETDRS measurement were taken into account at first visit and after 12, 24, 36, 48, and 60 months if available. For these visits, atrophy, scotoma points, and macular sensitivity were differentiated by foveal regions and longitudinal development was analyzed. For all missing measurement points, the data were statistically imputed. The availabilities of the measurements were 100% (20 study eyes) at the index time, 80% (16 study eyes) after 12 months, 85% (17 study eyes) after 24 months, 60% (12 study eyes) after 36 months, 15% (3 study eyes) after 48 months, and 20% (4 study eyes) after 60 months. 

Fellow eyes not meeting the criteria were also considered for specific analyses (regarding baseline characteristics and disease status; e.g., visual impairment was assessed for both eyes). Study eyes and fellow eyes were summarized as patient eyes for outcome analysis.

### 2.2. Characteristics of Participants

#### 2.2.1. Inclusion Criteria

The included participants comprised the following:

- Patients diagnosed with GA in one or both eyes as determined by the treating physician.
- Patients with total GA lesion areas of ≥2.5 and ≤17.5 mm^2^ (1 and 7 disc areas [DAs], respectively) imaging in at least one eye.
- If GA was multifocal, patients with at least one focal lesion of ≥1.25 mm^2^ and an overall aggregate GA area as described in the previous criterion.
- Patients with available MAIA microperimetric data at a minimum of 2 measurements with a timeframe of at least 2 years between the first and the last measurement.
- Patients aged ≥55 years at the index event.

#### 2.2.2. Exclusion Criteria

The exclusion criteria were as follows:

- Spherical equivalent of the refractive error demonstrating >6 diopters of myopia or an axial length >26 mm.
- Any history or active MNV, associated with AMD or any other cause, including any evidence of retinal pigment epithelium rips or evidence of neovascularization anywhere based on SD-OCT imaging or fluorescein angiography.
- Presence of an active ocular disease that, in the opinion of the treating physician, compromised or confounded visual function, including but not limited to uveitis, other macular diseases (e.g., clinically significant epiretinal membrane, full-thickness macular hole), or uncontrolled glaucoma/ocular hypertension (benign conditions in the opinion of the treating physician, such as peripheral retina dystrophy, were not exclusionary).
- Intraocular surgery (including lens replacement surgery) within 3 months prior to index event.
- Any history of laser therapy in the macular region.
- Aphakia or absence of the posterior capsule.
- Any ocular condition other than GA that may require surgery or medical intervention during the study period or, in the opinion of the treating physician, could compromise visual function during the study period.
- Any contraindication to intravitreal injection including current ocular or periocular infection.
- History of prior intravitreal injection.

### 2.3. Macular Integrity Assessment

**Figure 1:**
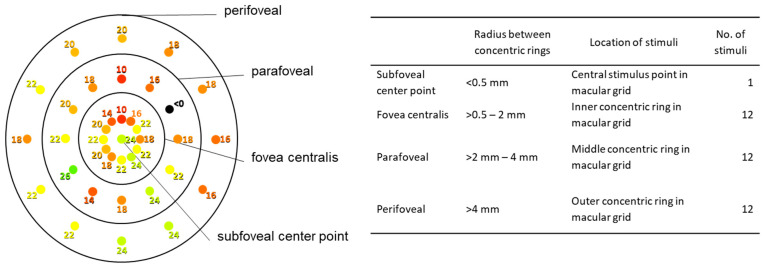
MAIA grid used in the present study.

Laser-stabilized macular microperimetry allows the overlaying of visual field data on the fundus image and has been established as a psychophysical method for assessment of localized central field sensitivity and thus for the detection of functional changes in AMD [23,24]. Microperimetry was performed by MAIA technology, which uses the standard MAIA macular grid (512 × 128 cube scan, centered over the macula) consisting of 37 stimuli: three concentric rings, each with 12 stimulus points, are arranged around the central foveal stimulus point, covering, in total, the central 10° (5° each side) of the macula (Figure 1). Stimulus rings are classified according to their positions relative to the central stimulus point as fovea centralis (innermost ring), parafoveal (middle ring), and perifoveal (outer ring). The latter two are collectively referred to as extrafoveal. Each stimulus point is activated, on average, 4–5 times at varying light intensities ranging from 0 dB (= brightest possible light intensity) to 36 dB (= least bright light intensity). Depending on the patient’s response to each stimulus, the light intensity of the next stimulus is varied by a 4-2 staircase projection strategy.

### 2.4. Baseline Variables

Baseline variables extracted from the medical charts included patient characteristics (age, gender, ethnicity), general ocular characteristics (lens status, intraocular pressure), GA characteristics (date of GA diagnosis, laterality, focality, localization, size and number of GA lesions), presence of double-layer signs (DLSs), central subfield thickness (CST), number and size of drusen (assessed by color fundus photography or scanning laser ophthalmoscopy), and presence of pseudodrusen (assessed by near-infrared reflectance imaging), visual impairment measures (NL-BCVA in ETDRS letters, comprising levels of visual impairment, as defined below), and scotoma characteristics (presence, type and number of scotoma, overall and stratified by localization relative to the fovea, assessed by MAIA).

### 2.5. Outcomes

GA lesions were evaluated for focality, localization, lesion size, and subfoveal encroachment (SFE). The definition of SFE was based on absolute scotoma, i.e., the lack of response to a 0 dB stimulus and, consequently, macular sensitivity of <0 dB as assessed by microperimetry. In case of presence of an absolute scotoma at the central stimulus point or at a stimulus point of the inner concentric ring (fovea centralis), GA lesion with involvement of the foveal center and thus SFE was assumed. This approach was chosen as fixation inaccuracies are common; if only the central stimulus point had been considered, false non-identification of actual SFE would have been very likely.

For evaluation of the robustness of the results with respect to the definition of SFE (presence of an absolute scotoma at the central stimulus point or at a stimulus point of the inner concentric ring), a sensitivity analysis was additionally performed with an alternative definition. Here, patients with SFE were required to present with an absolute scotoma at the central stimulus point and at a stimulus point of the inner concentric ring.

Visual impairment levels were defined for each eye as follows: no visual impairment as ≥70 ETDRS letters, mild impairment as <70 to ≥60 ETDRS letters, moderate impairment as <60 to ≥35 ETDRS letters, severe impairment as <35 to ≥20 ETDRS letters, blindness as per World Health Organization (WHO) definition as <20 to ≥5 ETDRS letters [25], and blindness as per German definition as <5 ETDRS letters [26].

### 2.6. Statistical Analysis

Upon completion of data extraction from the medical charts by the study site, the dataset was transferred in Microsoft Excel for data analysis. To prevent bias due to missing measurements and dropouts, imputation by linear mixed models was performed. For continuous variables, the sample size (n), mean, standard deviation (SD), minimum, median, and maximum were presented. For categorical variables, counts and percentages were determined. Results were obtained at first visit and after 12, 24, 36, 48, and 60 months. Results related to absolute scotoma and SFE analyses were presented stratified with respect to their relative location to the central stimulus point. In addition, time to event was analyzed from index to the development of SFE (only considering eyes at risk without initial SFE) and from index to the first increase in visual impairment. The statistical evaluation was performed using R 4.1.3 and Python 3.9.12.

## 3. Results

### 3.1. Patient Characteristics and Study Eyes

A total of 13 patients with 20 study eyes were included. The mean age was 69.7 years (57 to 84 years old). A total of 38.5% (n = 5) were male and 61.5% (n = 8) female. The ethnicity of all patients was categorized as white. In 53.8% (n = 7), both eyes were classified as study eyes, while for 46.2% (n = 6), only one eye was classified as a study eye. At the time of the index event, 50.0% (n = 10) of study eyes had 0-5 intermediate or large drusen (diameter ≥63 μm) while 35.0% (n = 7) had >20 drusen. Pseudodrusen were present in 20.0% (n = 4) of study eyes. A DLS was present in 10.0% (n = 2). The mean CST was 236.6 μm, ranging from 177.0 to 294.0 μm (median: 242.0 μm).

### 3.2. GA Lesions

**Figure 2:**
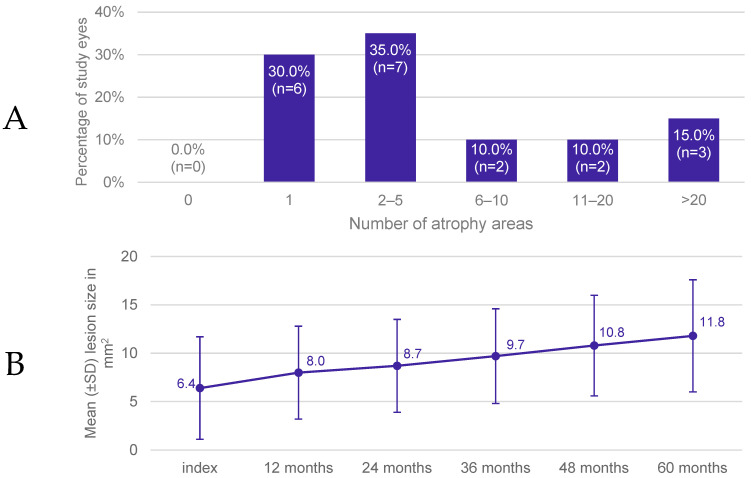
Development of GA lesions. (**A**) Number of atrophy areas of study eyes at time of index event and (**B**) mean (±SD) GA lesion size assessed by SD-OCT over time.

At the time of the index, 92.3% (n = 12) of patients already showed bilateral GA. Notably, due to the application of inclusion and exclusion criteria, only for 53.8% (n = 7) of these patients, both eyes were classified as study eyes. Atrophy areas were unifocal in 30.0% (n = 6) and multifocal in 70.0% (n = 14) of study eyes. A total of 35.0% (n = 7) of study eyes had 2–5 multifocal lesions and 15.0% (n = 3) had >20 atrophy areas (Figure 2A).

At index, the mean overall GA lesion size assessed by SD-OCT was 6.4 mm^2^ (SD: 5.3 mm^2^), ranging from 0.2 to 15.0 mm^2^ (median: 3.9 mm^2^). The overall mean square root of the GA lesion size was 2.3 mm (SD: 1.1 mm), ranging from 0.4 to 3.9 mm (median: 2.0 mm). During the 60 months of follow-up, the mean overall lesion size increased by 5.4 mm^2^ to 11.8 mm^2^ (1.08 mm^2^/year) (Figure 2B). Of note, the respective results were influenced by two outliers. The exclusion of the outliers resulted in a more pronounced observable increase in the mean overall lesion size of 6.7 mm^2^ (1.34 mm^2^/year; from 5.8 mm^2^ to 12.5 mm^2^). A maximum GA lesion size of 20.9 mm^2^ at 60 months was observed.

At the time of the index, 30.0% (n = 6) of study eyes already showed SFE while 70.0% (n = 14) showed none. Of those 14 eyes at risk, 78.6% (n = 11) developed SFE after an average time of 2.9 years (SD: 1.8 years). The sensitivity analysis of SFE (presence of an absolute scotoma at the central stimulus point and at least one stimulus point of the inner concentric ring) revealed that 10.0% (n = 2) of the study eyes had SFE at the time of the index while in 27.8% (n = 5) of the remaining 18 eyes at risk, SFE was observed after an average period of 2.4 years (SD: 1.9).

In the case of bilateral GA, the average time to the first documentation of SFE was 1.9 years (SD: 1.4 years) in the worse-seeing and 3.4 years (SD: 2.0 years) in the better-seeing eyes. All (n = 6) better-seeing study eyes at risk developed SFE during the observation time.

### 3.3. Visual Impairment

**Figure 3:**
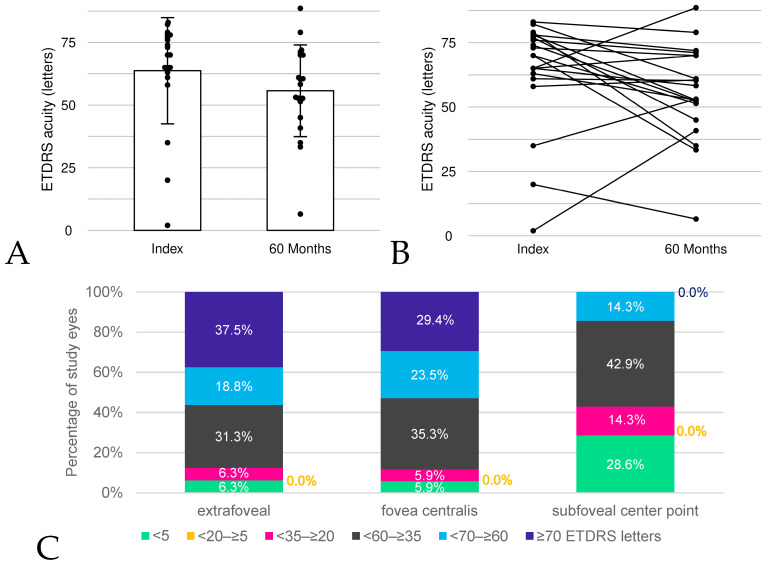
Development of NL-BCVA over time and at first occurrence of absolute scotoma points, assessed by ETDRS letters. Within 60 months, mean NL-BCVA decreased by 8 letters (**A**). Development for each individual patient is shown in (**B**). In some cases, a positive trend emerged due to individual variance and imputation with few data points. Visual impairment was assessed at first occurrence of absolute scotoma points by localization relative to the fovea (**C**).

At the index event, the mean NL-BCVA of the study eyes was 63.7 ETDRS letters, ranging from 2.0 to 83.0 letters (median: 70.0). Within 60 months, mean NL-BCVA decreased by 8 letters (−1.6 letters/year on average) from 63.7 (SD: 21.2) to 55.7 (SD: 18.3; Figure 3A,B).

Following the above-described classification of visual impairment, 55.0% (n = 11) of the study eyes had no visual impairment (≥70 ETDRS letters) at the time of the index, 40.0% (n = 8) had mild to severe visual impairment (<70–≥20 ETDRS letters), and one study eye (5.0%) was blind as per the German legal definition. During the follow-up, the percentage of eyes in the no-visual-impairment category decreased by 25 percentage points to 30.0% (n = 6) at 60 months. In contrast, the proportion of study eyes with mild to severe visual impairment increased to 65.0% (n = 13) at 60 months. The average time from the index to the first increase in visual impairment level was 1.7 years (SD: 0.9 years).

In addition, visual impairment levels were assessed at the time of the first observation of an absolute scotoma point at the central stimulus point, the fovea centralis, and the extrafoveal region (Figure 3C). If an absolute scotoma point already existed in the respective region at the time of the index, the index date was considered. At the time of the occurrence of the first extrafoveal absolute scotoma point, 37.5% (n = 6) of study eyes were in the no-visual-impairment category while only 29.4% (n = 5) of study eyes showed no visual impairment when the fovea centralis was affected by absolute scotoma. Once a scotoma was measured at the subfoveal center point, all study eyes displayed some kind of visual impairment, with none remaining in the ‘no visual impairment’ category. Visual impairment category <5 ETDRS letters (blindness occurring according to the German legal definition) accounted for 6.3% (n = 1) of the study eyes at the time of the first absolute scotoma point being detected in the extrafoveal area but 28.6% (n = 2) of the study eyes when detected at the subfoveal center point. In conclusion, the encroachment of the absolute scotoma in the very center of the fovea severely impacts visual acuity. The proportion of study eyes in the NL-BCVA category ≥70 ETDRS letters decreased from 72.7% at the last measurement prior to SFE to 7.1% at the first measurement after SFE. Applying the SFE alternative definition used in the sensitivity analysis, visual impairments were detected in all study eyes at and after SFE and 43.1% (11.8% using the definition of the main analysis) of the study eyes had at least severe visual impairment at the time of the first measurement of SFE.

### 3.4. Absolute Scotoma: Longitudinal Development

At the time of the index, at least one absolute scotoma point (<0 dB; based on the 37 MAIA stimulus points) was present in 45.0% (n = 9) of the study eyes. Among those, 35.0% (n = 7) of the study eyes had perifoveal, 45.0% (n = 9) parafoveal, 30.0% (n = 6) foveal, and 10.0% (n = 2) central subfoveal absolute scotoma points. 

**Figure 4:**
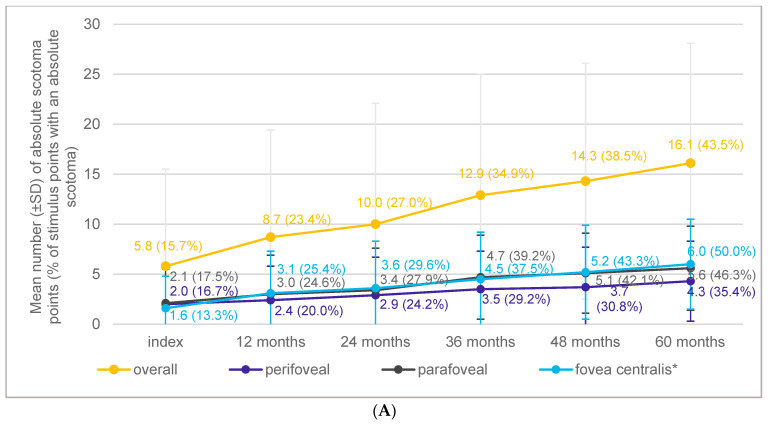

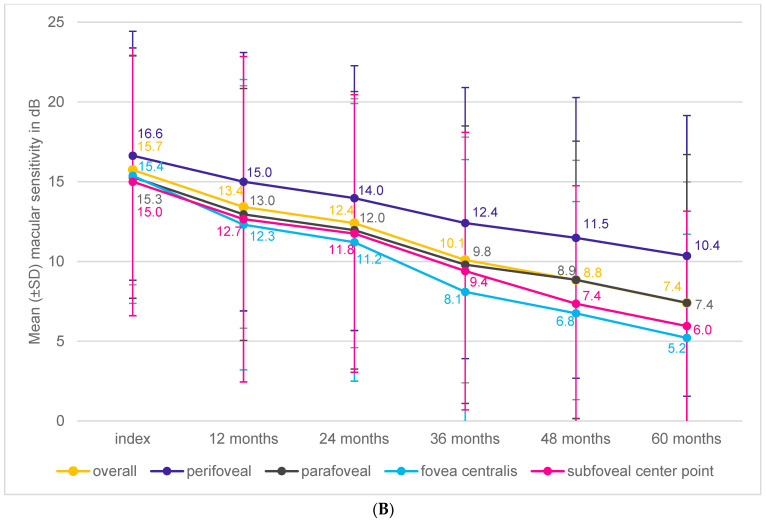
Development of absolute scotoma points and change in macular sensitivity over time by localization relative to the fovea. The mean number of absolute scotoma points increased by 10.3 (29.1%) within 60 months (>2 absolute scotoma points/year (**A**) while the mean overall macular sensitivity decreased by 8.3 dB after 60 months (−0.6 dB/year) (**B**). * Fovea centralis was defined as the inner concentric ring in macular grid (>0.5–2 mm, 12 stimuli IDs) without the subfoveal center point.

The mean number of absolute scotoma points at the index event was 5.8 (SD: 9.7; 15.7% of stimulus points), ranging from 0.0 to 37.0 (median: 0.0). The mean number of absolute scotoma points increased by 10.3 (29.1%) from 5.8 (SD: 9.7; 15.7%) to 16.1 (SD: 12.0; 43.5%) after 60 months (>2 absolute scotoma points/year; Figure 4A). A total of 15.0% (n = 3) of the study eyes did not experience any increase in absolute scotoma point during follow-ups.

The mean number of annual new immediately adjacent absolute scotoma points (new absolute scotoma points located 500 μm around the respective primary scotoma point) increased from 1.6 (SD: 4.1) at 12 months to 1.9 (SD: 2.5) at the 60-month follow-up.

### 3.5. Macular Sensitivity

The mean overall macular sensitivity at the index event was 15.7 dB, ranging from −1.0 to 25.6 dB (median: 18.8 dB), and decreased by 8.3 dB to 7.4 dB after 60 months (−0.6 dB/year; Figure 4B). By localizations, the strongest decrease of about 10 dB from 15.4 dB to 5.2 dB was detected within the fovea centralis. Overall, the mean macular sensitivity was 18.1 dB at no visual impairment and decreased with each worse visual impairment level to 1.8 dB at blindness as per the German legal definition. At the subfoveal center point, average macular sensitivity was 16.8 dB with no visual impairment and −1.0 with blindness as per the German definition.

## 4. Discussion

### 4.1. Key Results

**Figure 5:**
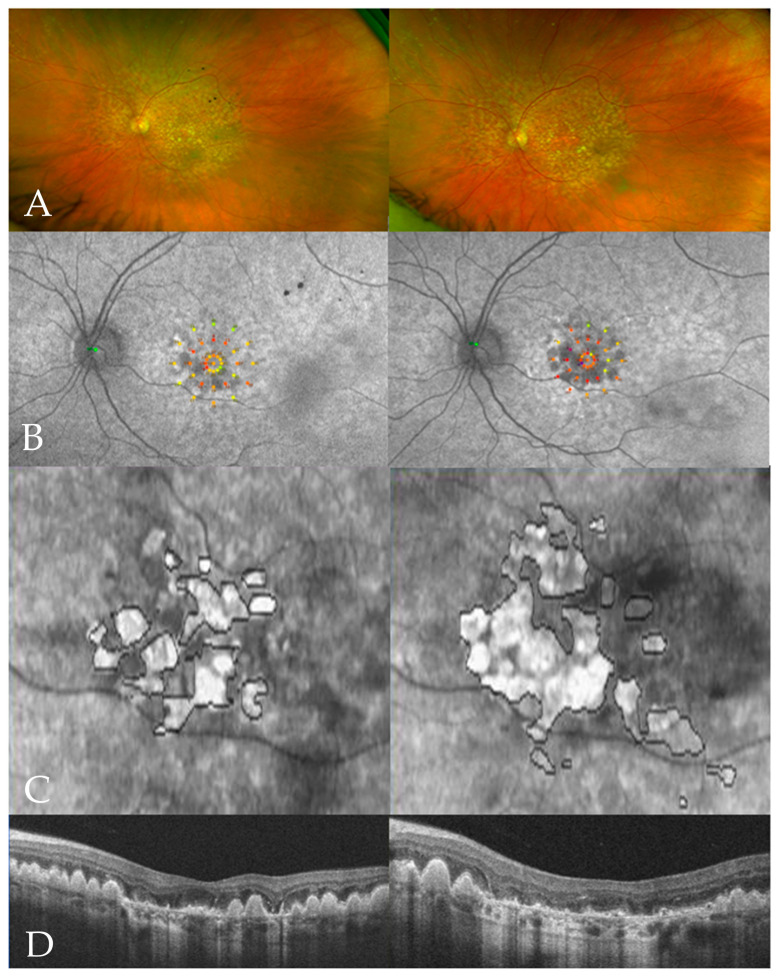
Representative SLO, MAIA microperimetry, and SD-OCT data of the left eye in a patient with GA at the first presentation (left half) and after 60 months (right half). SLO shows an overall increase in the number of drusen, both centrally and peripherally, as well as the growth of central atrophy areas (**A**). The MAIA grid superimposed on the OCT scan shows the corresponding functional decline in macular sensitivity (**B**). Spread of the atrophy areas as mapped in the en face (**C**) and B-scan OCT (**D**).

The study results were a documentation of the structural and functional aspects of the natural progression of GA (Figure 5). The strict inclusion criteria led to an exclusive assessment of patients with isolated GA. Both neovascular AMD as well as other severe ocular diseases and ocular surgery within 3 months prior to the index event were excluded. Challenges due to a relatively low study sample size were offset by a comprehensive long-term follow-up. The first cause of disease progression is the expansion of RPE degeneration and, consequently, the spread of atrophic lesions from the peripheral macula towards the center [10]. The mean GA lesion size increased by 5.4 mm^2^ from 6.4 mm^2^ to 11.8 mm^2^ within 60 months (1.08 mm^2^/year) and was even more pronounced (from 5.8 mm^2^ to 12.5 mm^2^; 1.34 mm^2^/year) when excluding outliers, which corresponded to the increase rates reported in the literature (from 0.53 to 2.8 mm^2^/year) [9,27]. There was a lower growth in lesion size observed in this study compared to the DERBY and OAKS clinical trials. This can probably be explained due to the different lesion sizes at baseline (6.4 mm^2^ vs. 8.25 mm^2^) [9,18]. A total of 78.6% of study eyes not centrally affected at the baseline developed SFE of the lesions during follow-up after an average of 2.9 years. During this period of ‘foveal sparing’, central vision was relatively well preserved [11].

Defined as spots of light insensitivity, the occurrence of absolute scotoma points showed an average increase in number from 5.8 (15.7% of stimulus points) at the baseline to 16.1 (43.5% of stimulus points) after 60 months. 

In a slow but steady process, the overall mean NL-BCVA decreased from 63.7 to 55.7 ETDRS letters within 60 months. The proportion of eyes without visual impairment was reduced from 55.0% to 30.0%. As is to be expected, the extent of foveal involvement is critical for the extent of visual impairment. The proportion of study eyes without visual impairment decreased by about 65.0% after SFE was detected. Even more notable, when applying the alternative definition for SFE (sensitivity analysis), all study eyes showed visual impairment after SFE and over 40.0% had at least severe visual impairment.

**Figure 6:**
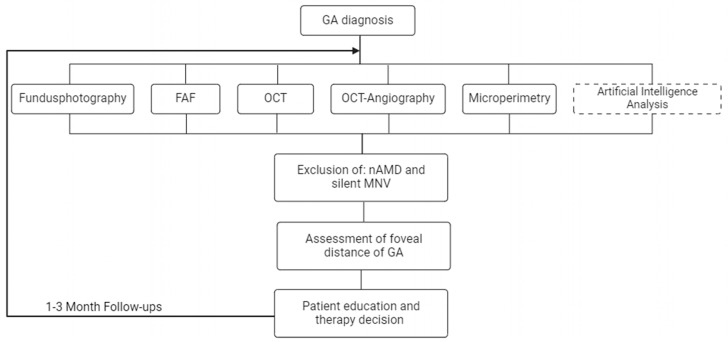
Flow chart for the diagnosis and monitoring of GA. In addition to patient education and exclusion of nAMD, management of GA should include broad diagnostic monitoring.

The methods applied and related in this study led to results that blend in well with the existing knowledge of the natural history of GA. These data should contribute to the completion of the picture of the natural disease course of GA in patients of a German medical center, which can serve as reference for future therapeutic approaches. For present clinical application, the data provide a context for the monitoring of GA progression (Figure 6). The findings available here were pioneers for corresponding statistical analyses in the OAKS study, which showed a functional benefit for pegcetacoplan. The protection of the fovea and the prevention of the development of central scotoma points in the context of an evaluation of GA therapies will probably become more of a central focus in the future and will need to be discussed more intensively.

### 4.2. Comparison to the Clinical Trial OAKS

Both the inclusion criteria as well as the selection of the variables were based on the phase 3 clinical trial OAKS [21], allowing a certain comparability of our results with this much more extensive study (614 included patients). Patients in the present study were on average 8.8 years younger than patients in the OAKS trial. The initial GA lesion size was 6.4 mm^2^ in our study vs. 8.2 mm^2^ in OAKS, with a proportion of 30.0% initial SFE in our study vs. 64.1% in OAKS. Pseudodrusen were present in 20.0% of our study eyes vs. 84.4% in OAKS while a DLS was present in 10.0% of our study eyes vs. 17.3% in OAKS. Other variables like the course of NL-BCVA, GA focality, and laterality did not differ substantially. Overall, the OAKS study observed a pool of patients at a more advanced stage of GA.

The MAIA grid used in this study, as described above, differs from the 10-2 MAIA grid applied in OAKS, which covered 10 central degrees on each side (in total, a central 20 degrees) and comprised 68 stimulus points arranged like a chessboard with a distance of 2 degrees (about 500–600 μm) between each pair of stimulus points without assessing a central stimulus point. However, the comparability of the change in measurements can be assumed.

### 4.3. Main Limitations of the Study

As only one study site participated in this study, the representativeness and generalizability of the results might be limited. The data quality depends on the completeness and accuracy of the medical charts, both in terms of documented functional patient data and FAF and OCT(-A) image reading, whereby FAF measurements were not undertaken for all patients. Not all patient charts contained the information that was required to address the study objectives, which was why imputation for missing data points was performed by linear mixed models. MAIA measurements were assumed to be centrally located over the anatomical foveal location. Regarding the definition of SFE, an approximation via the presence of absolute scotoma points in the absolute center point and/or the inner ring of the MAIA grid was applied. If only the absolute central point were considered, the fixation could shift in approximately 25–30% of cases. For this reason, we refrained from limiting the approximation only to the absolute central stimulus point. Overall, the assessment of SFE could only be considered a best estimate based on the available data since OCT imaging displaying the lesion coverage of the absolute center of the fovea, unlike in the OAKS study, was not available.

## 5. Conclusions

Overall, the analyses of GA lesion sizes, NL-BCVA, and scotoma points showed worsening trends during the 60 months of follow-up. The development of central absolute scotoma was a key factor for the increasing deterioration of macular outcomes, an observation that has also been observed in analyses from the phase 3 study OAKS comparing pegcetacoplan to sham in patients with GA. Absolute scotoma in the center of the fovea has a crucial impact on visual function and, therefore, its avoidance is highly relevant for patients. The study results were a documentation of the longitudinal structural and functional outcomes of the macula in patients with mostly isolated GA. Due to the strict orientation based on the OAKS study criteria, the identified patient pool would be suitable for potential future treatment with intravitreal complement inhibitors.

## 6. Supplementary Information

Supplementary to the findings presented in the main part of the manuscript, further outcomes in terms of relative scotoma points (presence and number of scotoma, overall and stratified by localization relative to the fovea, assessed by MAIA) and fixation stability (overall and stratified by localization, assessed by MAIA in °^2^ at 63% and 95% BCEA) were assessed in the course of the study.

Relative scotoma was stratified by responses to stimuli between 0 dB and ≤5 dB, 0 dB and ≤15 dB, and 0 dB and ≤25 dB [28]. Fixation stability was measured in degrees squared (°^2^) at both 63% and 95% confidence intervals as determined by BCEA [20], wherein an increase signified a loss of fixation stability. 

### 6.1. Relative Scotoma

At the index, all study eyes (100.0%) had at least one scotoma point (absolute and/or relative scotoma) based on ≤25 dB, being localized at the subfoveal center point in 95.0% (n = 19) of cases. Based on ≤15 dB, this proportion decreased to 85.0% (n = 17), with 35.0% (n = 7) at the subfoveal center point. Based on ≤5 dB 60.0% (n = 12) remained, with 20.0% (n = 4) at the subfoveal center point.

**Figure 7:**
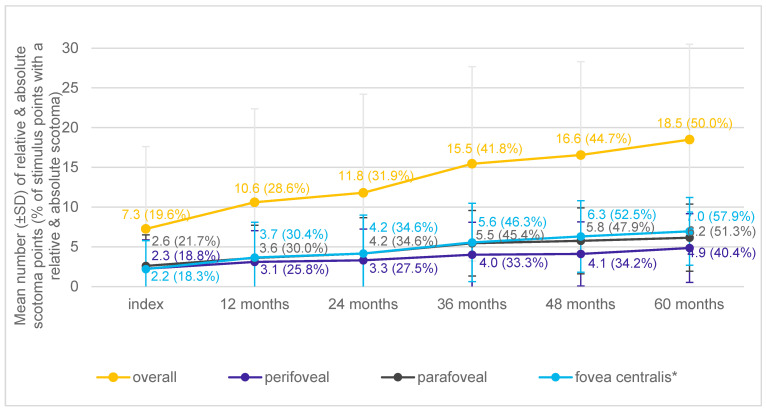
Development of mean number (±SD) of absolute and/or relative scotoma points over time based on ≤5 dB by localization relative to the fovea. * Fovea centralis was defined as the inner concentric ring in macular grid (>0.5–2 mm, 12 stimuli IDs) without the subfoveal center point.

The mean number of overall scotoma points was 31.7, based on ≤25 dB (range: 6.0–37.0; median: 34.0), and increased to 34.0 after 60 months. Based on ≤15 dB, there were 13.1 overall scotoma points (range: 0.0–37.0; median: 7.5), increasing to 25.7, and based on ≤5 dB, there were 7.3 (range: 0.0–37.0; median: 1.5), increasing to 18.5 after 60 months of follow-up (see Figure 7 for scotoma ≤5 dB).

### 6.2. Fixation Stability

The mean baseline fixation stability at 63% BCEA was 5.9°^2^, ranging from 0.1°^2^ to 32.2°^2^ (median: 2.8°^2^), and at 17.6°^2^, 95% BCEA, ranging from 0.4°^2^ to 96.5°^2^ (median: 8.5°^2^). Within 60 months, the areas increased by 2.1°^2^ from 5.9°^2^ to 8.0°^2^ at 63% BCEA and by 6.5°^2^ from 17.6°^2^ to 24.1°^2^ at 95% BCEA, showing losses in fixation stability (0.44°^2^/year and 1.3°^2^/year, respectively).

**Figure 8:**
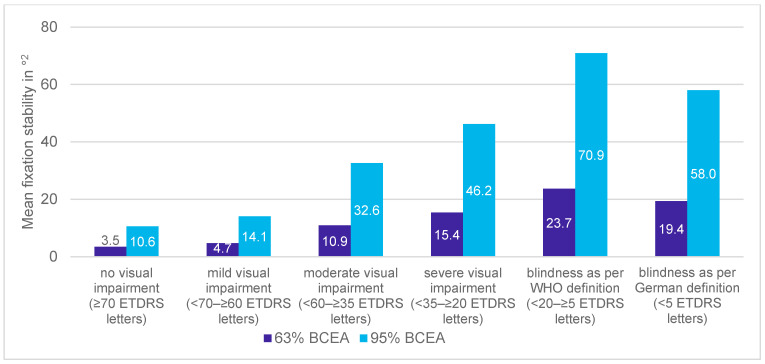
Mean fixation stability at 63% and 95% BCEA at the time of first documentation of each visual impairment level.

At the stage of no visual impairment, the mean fixation stability was 3.5°^2^ at 63% and 10.6°^2^ at 95% BCEA and declined to 23.7°^2^ at 63% and 70.9°^2^ at 95% BCEA at the stage of blindness as per the WHO (Figure 8).

## Data Availability

The datasets generated and analyzed during the current study are not publicly available but are available from the corresponding author on reasonable request.

## Abbreviations

AMD	Age-related macular degeneration
BCEA	Bivariate contour ellipse area
CST	Central subfield thickness
DA	Disc areas
DLS	Double-layer sign
ETDRS	Early Treatment Diabetic Retinopathy Study
FAF	Fundus autofluorescence
GA	Geographic atrophy
I.I.O.	Internationale Innovative Ophthalmochirurgie GbR
MAIA	Macular integrity assessment
n	Sample size
NL-BCVA	Normal-luminance best-corrected visual acuity
OCT-A	Optical coherence tomography angiography
RPE	Retinal pigment epithelium
SD	Standard deviation
SD-OCT	Spectral-domain optical coherence tomography
SFE	Subfoveal encroachment
SLO	Scanning Laser Ophtalmoscopy
WHO	World Health Organization
